# Supplementary material for: Efficient perpendicular magnetization switching by a magnetic spin Hall effect in a noncollinear antiferromagnet
Source: Nat Commun. 2022 Aug 1;13:4447. doi: 10.1038/s41467-022-32179-2 (PMC9343665; doi:10.1038/s41467-022-32179-2)
Supplement: Supplementary file 1 — Supplementary Information [file 41467_2022_32179_MOESM1_ESM.docx]

**Supplementary Information**

Efficient perpendicular magnetization switching by a magnetic spin Hall effect in a noncollinear antiferromagnet

Shuai Hu^1†^, Ding-Fu Shao^2,3†^, Huanglin Yang^1†^, Chang Pan^1†^, Zhenxiao Fu^4,5^, Meng Tang^1^, Yumeng Yang^4,5*^, Weijia Fan^1^, Shiming Zhou^1^, Evgeny Y. Tsymbal^2*^ and Xuepeng Qiu^1*^

*^1^Shanghai Key Laboratory of Special Artificial Microstructure Materials and Technology and School of Physics Science and Engineering, Tongji University, Shanghai 200092, China*

*^2^Department of Physics and Astronomy and Nebraska Center for Materials and Nanoscience, University of Nebraska, Lincoln, Nebraska 68588-0299, USA*

*^3^ Key Laboratory of Materials Physics, Institute of Solid State Physics, HFIPS, Chinese Academy of Sciences, Hefei 230031, China*

*^4^School of Information Science and Technology, ShanghaiTech University, Shanghai 201210, China*

*^5^Shanghai Engineering Research Center of Energy Efficient and Custom AI IC, School of Information Science and Technology, ShanghaiTech University, Shanghai 201210, China*

**Supplementary Note 1: Symmetry and electronic properties of Mn_3_Sn**

**Supplementary Note 2**: **Epitaxial deposition and characterization of Mn_3_Sn thin film**

**Supplementary Note 3**: **Signature of Weyl magnet by magneto-transport measurements**

**Supplementary Note 4**: **Perpendicular magnetic anisotropy of Mn_3_Sn and *β*-Ta based heterostructure device**

**Supplementary Note 5: Estimation of the out-of-plane polarized spin current by MSHE**

**Supplementary Note 6**: **Exchange bias effect of Mn_3_Sn based heterostructure**

**Supplementary Note 7**: **SOT switching results for I // H // Mn_3_Sn**$\boldsymbol{[2}\bar{\boldsymbol{1}}\bar{\boldsymbol{1}}\boldsymbol{0]}$ **and I// Mn_3_Sn**$\left[ \boldsymbol{01}\bar{\boldsymbol{1}}\boldsymbol{0} \right]$**, H // Mn_3_Sn**$\boldsymbol{[2}\bar{\boldsymbol{1}}\bar{\boldsymbol{1}}\boldsymbol{0]}$

**Supplementary Note 8**: **Evaluation of Joule heating effect and other control experiments**

**Supplementary Note 9. Magnetization switching by MSHE in a 100 nm device**

**Supplementary Note 10: Macro-spin simulation of MSHE driven magnetization switching**

**Supplementary Note 11: Thermal effects during MSHE driven magnetization switching**

**Supplementary Note 1: Symmetry and electronic properties of Mn_3_Sn**

Mn_3_Sn has a hexagonal Ni_3_Sn-type structure with space group *P*6_3_/*mmc*^1^. Below the Néel temperature *T*_N_ ~ 420 K, there are two types of the inverse triangular noncollinear antiferromagnetic orders, i.e. AFM1 and AFM2 (Fig. 1a) in Mn_3_Sn^2-4^. AFM1 has the magnetic space group *cmc’m’*, which contains a mirror symmetry $M_{x}$ perpendicular to the *x* ($[01\overline{1}0]$) direction. This symmetry allows finite canting of magnetic moments as schematically shown in Fig. 1a, resulting in a net magnetization along the *x* direction. AFM2 has the magnetic space group *cm’cm’* containing a glide symmetry $G_{y}=\{M_{y}|\frac{c}{2}\}$ (a mirror operation plus a half-unit-cell translation along the [0001] direction) perpendicular to the *y* ($[2\overline{11}0]$) direction. This symmetry allows a net magnetization along the *y* direction (Fig. 1a). Therefore, the AFM1 and AFM2 states can be switched by reorienting the net magnetization using an in-plane magnetic field^2^.


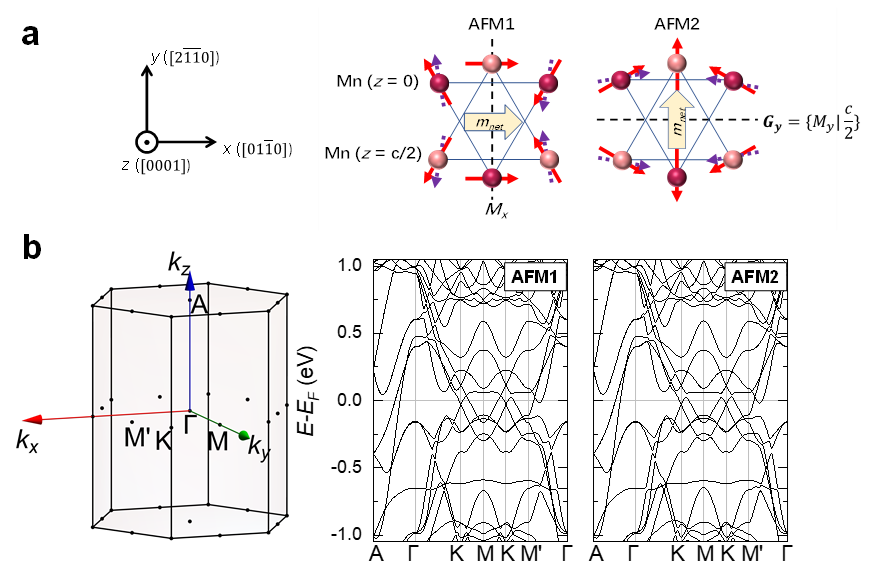


**Figure 1**: **a** The magnetic configurations of AFM1 and AFM2 of Mn_3_Sn. The solid arrows denote the ideal magnetic moments, while the dashed arrows denote the canted moments allowed by symmetry. This symmetry allows finite canting of magnetic moments as schematically shown in Fig. 1a, resulting in net magnetization along the x(y) direction for AFM1(2). **b** The calculated band structures of AFM1 and AFM2 of Mn_3_Sn.

The different symmetries result in different electronic properties of AFM1 and AFM2. Figure 1(b) shows the band structures of these two states obtained by first-principles DFT calculations, which are consistent to those for AFM1^5^ and for AFM2^6,7^ calculated previously. The symmetry related differences in the band structures can be seen from Figure 1b for these two states.

Table 1 shows the tensors of spin Hall conductivity (SHC) and magnetic spin Hall conductivity (MSHC) resulting from the AFM1 and AFM2 symmetries. An out-of-plane anti-damping torque for the efficient switching of perpendicular magnetization is supported by the conductivity components $\sigma_{zx}^{z}$ and $\sigma_{zy}^{z}$, which are vanishing in SHC but allowed for MSHC. Specifically, we find that AFM1 exhibits finite $\sigma_{zx}^{z}$ and vanishing $\sigma_{zy}^{z}$, while AFM2 exhibits finite $\sigma_{zy}^{z}$ and vanishing $\sigma_{zx}^{z}$, due to different symmetries. This is correctly reflected by our calculation in Fig. 1d in the main text, and consistent with our experimental observations.

**Table 1**: The tensors of spin Hall conductivity (SHC) and magnetic spin Hall conductivity (MSHC) for AFM1 and AFM2 Mn_3_Sn.

|  |  |  | $\boldsymbol{\sigma}^{\boldsymbol{x}}$ | $\boldsymbol{\sigma}^{\boldsymbol{y}}$ | $\boldsymbol{\sigma}^{\boldsymbol{z}}$ |
| --- | --- | --- | --- | --- | --- |
|  |  | **General tensor** | $\left[ \begin{matrix} \sigma_{xx}^{x} & \sigma_{xy}^{x} & \sigma_{xz}^{x} \\ \sigma_{yx}^{x} & \sigma_{yy}^{x} & \sigma_{yz}^{x} \\ \sigma_{zx}^{x} & \sigma_{zy}^{x} & \sigma_{zz}^{x} \end{matrix} \right]$ | $\left[ \begin{matrix} \sigma_{xx}^{y} & \sigma_{xy}^{y} & \sigma_{xz}^{y} \\ \sigma_{yx}^{y} & \sigma_{yy}^{y} & \sigma_{yz}^{y} \\ \sigma_{zx}^{y} & \sigma_{zy}^{y} & \sigma_{zz}^{y} \end{matrix} \right]$ | $\left[ \begin{matrix} \sigma_{xx}^{z} & \sigma_{xy}^{z} & \sigma_{xz}^{z} \\ \sigma_{yx}^{z} & \sigma_{yy}^{z} & \sigma_{yz}^{z} \\ \boldsymbol{\sigma}_{\boldsymbol{zx}}^{\boldsymbol{z}} & \boldsymbol{\sigma}_{\boldsymbol{zy}}^{\boldsymbol{z}} & \sigma_{zz}^{z} \end{matrix} \right]$ |
|  | **AFM1** | **SHC** | $\left[ \begin{matrix} 0 & 0 & 0 \\ 0 & 0 & \sigma_{yz}^{x} \\ 0 & \sigma_{zy}^{x} & 0 \end{matrix} \right]$ | $\left[ \begin{matrix} 0 & 0 & \sigma_{xz}^{y} \\ 0 & 0 & 0 \\ \sigma_{zx}^{y} & 0 & 0 \end{matrix} \right]$ | $\left[ \begin{matrix} 0 & \sigma_{xy}^{z} & 0 \\ \sigma_{yx}^{z} & 0 & 0 \\ 0 & 0 & 0 \end{matrix} \right]$ |
|  |  | **MSHC** | $\left[ \begin{matrix} \sigma_{xx}^{x} & 0 & 0 \\ 0 & \sigma_{yy}^{x} & 0 \\ 0 & 0 & \sigma_{zz}^{x} \end{matrix} \right]$ | $\left[ \begin{matrix} 0 & \sigma_{xy}^{y} & 0 \\ \sigma_{yx}^{y} & 0 & 0 \\ 0 & 0 & 0 \end{matrix} \right]$ | $\left[ \begin{matrix} 0 & 0 & \sigma_{xz}^{z} \\ 0 & 0 & 0 \\ \boldsymbol{\sigma}_{\boldsymbol{zx}}^{\boldsymbol{z}} & \boldsymbol{0} & 0 \end{matrix} \right]$ |
|  | **AFM2** | **SHC** | $\left[ \begin{matrix} 0 & 0 & 0 \\ 0 & 0 & \sigma_{yz}^{x} \\ 0 & \sigma_{zy}^{x} & 0 \end{matrix} \right]$ | $\left[ \begin{matrix} 0 & 0 & \sigma_{xz}^{y} \\ 0 & 0 & 0 \\ \sigma_{zx}^{y} & 0 & 0 \end{matrix} \right]$ | $\left[ \begin{matrix} 0 & \sigma_{xy}^{z} & 0 \\ \sigma_{yx}^{z} & 0 & 0 \\ 0 & 0 & 0 \end{matrix} \right]$ |
|  |  | **MSHC** | $\left[ \begin{matrix} 0 & \sigma_{xy}^{x} & 0 \\ \sigma_{yx}^{x} & 0 & 0 \\ 0 & 0 & 0 \end{matrix} \right]$ | $\left[ \begin{matrix} \sigma_{xx}^{y} & 0 & 0 \\ 0 & \sigma_{yy}^{y} & 0 \\ 0 & 0 & \sigma_{zz}^{y} \end{matrix} \right]$ | $\left[ \begin{matrix} 0 & 0 & 0 \\ 0 & 0 & \sigma_{yz}^{z} \\ \boldsymbol{0} & \boldsymbol{\sigma}_{\boldsymbol{zy}}^{\boldsymbol{z}} & 0 \end{matrix} \right]$ |

**Supplementary Note 2**: **Epitaxial deposition and characterization of Mn_3_Sn thin film**

The hexagonal antiferromagnet Mn_3_Sn exhibits a stacked Kagome lattice and inverse triangular magnetic order of Mn moments. An epitaxial Mn_3_Sn film can be directly grown on hexagonal Al_2_O_3_ $(1\bar{1}02)$ or cubic MgO (111) substrates, with a crystal orientation of $(11\bar{2}0)$ and (0001), respectively. Among them, Mn_3_Sn$(11\bar{2}0)$ typically possesses a relatively large AHE, which hinders the clear identification of the AHE signal from the ferromagnetic [Ni/Co]_3_ multilayers. On the contrary, the AHE of (0001) Mn_3_Sn is negligible and the perpendicular anisotropy of [Ni/Co]_3_ multilayer can be promoted on Mn_3_Sn (0001) through structural engineering. Therefore, in this study, the Mn_3_Sn is epitaxially deposited on the MgO (111) substrate by dc magnetron sputtering. Before proceeding to SOT study, the Mn_3_Sn film quality is examined by using X-ray diffraction technique, and the result of 2*θ* scan is shown in Fig. 2a. Except for those peaks from MgO substrates, the only discernable diffraction peaks are (0002) and (0004) from Mn_3_Sn. This confirms the absence of other phases within the measurement sensitivity.

**Figure 2: Crystal quality of epitaxial Mn_3_Sn thin film**. **a** *θ*-2*θ* XRD spectra of 40 nm thick Mn_3_Sn film and sole MgO (111) substrate using for comparison. **b** HR-TEM image of the Mn_3_Sn sample deposited on the MgO substrate. **c** HR-TEM image of the whole stack of Mn_3_Sn/Cu/[Ni/Co]_n_/Cu. **d** EDS mapping of Mn_3_Sn/Cu/[Ni/Co]_n_/Cu.

The sharp interface and excellent epitaxial quality are also supported by the high-resolution transmission electron microscopy (HR-TEM) image of the cross section of Mn_3_Sn (0001)/MgO (111) (Fig. 2b). In addition, the whole stack of Mn_3_Sn/Cu/[Ni/Co]_n_/Cu has been also carefully characterized. Figure 2c shows the HR-TEM image of the multilayers and good crystalline texture of both Mn_3_Sn and Cu/[Ni/Co]_n_ multilayer can be seen. Energy-dispersive X-ray spectroscopy (EDS) mapping has been also performed. As shown in Figure 2d, the EDS mapping result clearly resolves the elements layer by layer, as depicted using different colors. This result demonstrates sharp interfaces, small roughness, and negligible inter-layer diffusion between different layers.

We also characterize the magnetic properties of as-deposited film. The as-deposited Mn_3_Sn film exhibits a very soft magnetic hysteresis behaviour at room temperature (Fig. 3), indicating that its domains can be manipulated by an external magnetic field. With the thermal activation by Joule heating, when the film is patterned into a Hall bar device, the domains can be reoriented even in a field much smaller than the coercive field at room temperature.

**Figure 3**: Magnetic hysteresis loop for a 7 nm Mn_3_Sn film. The inset shows the amplified loop data at small magnetic fields.

**Supplementary Note 3**: **Signature of Weyl magnet by magneto-transport measurements**

Previous experimental evidences suggest that the existence of magnetic Weyl fermions in bulk Mn_3_Sn can be probed directly by the chiral anomaly activated anisotropic magnetoconductance from magneto-transport measurements^5^. To verify this property in our thin-film samples, micron-sized Hall bar devices are fabricated using standard photolithography techniques. As illustrated in Fig. 4a, the devices consist of two types with orthogonal longitudinal electrodes along $\left[ 01\bar{1}0 \right]$ and $[2\bar{1}\bar{1}0]$direction, respectively. Since the measurement results of these two types are similar, hereafter only the results from the $\left[ 01\bar{1}0 \right]$ aligned Hall bar devices are presented.


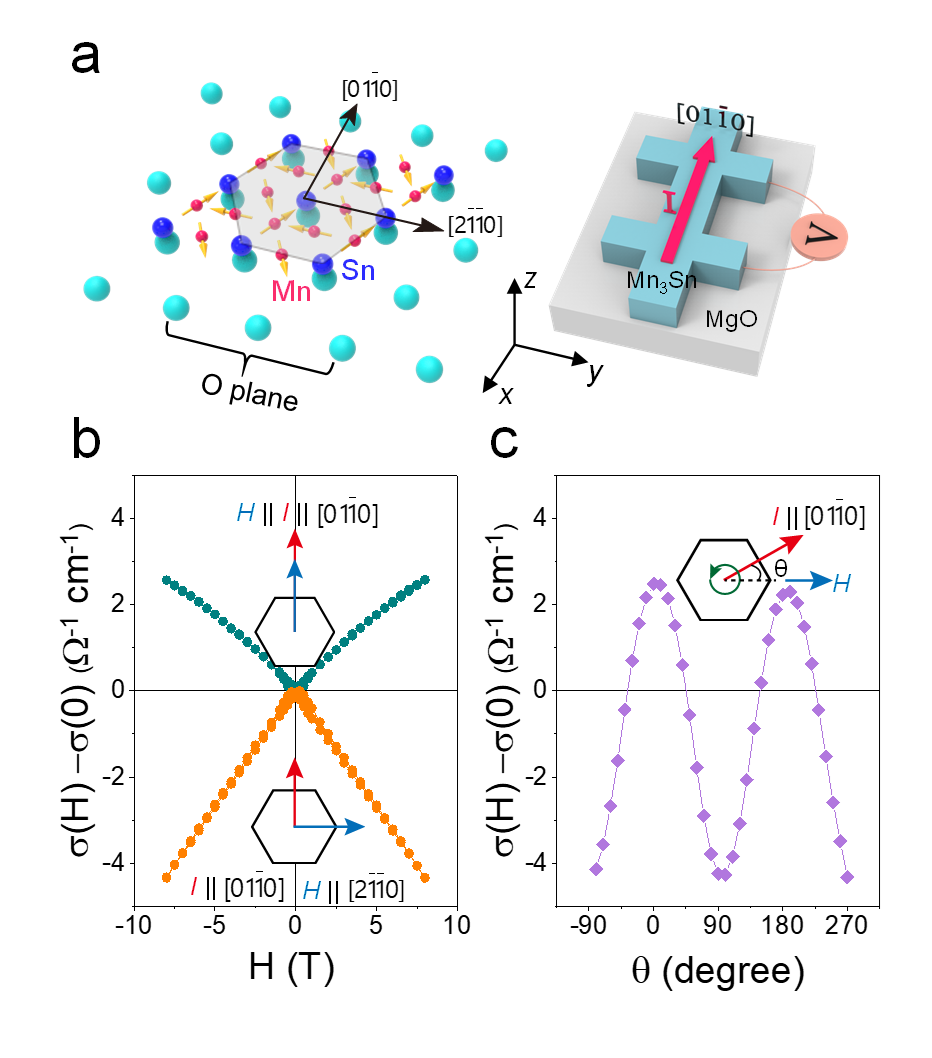


**Figure 4: Magneto-transport results of Mn_3_Sn Hall bar devices. a** Crystal and magnetic structure of the *a-b* plane of Mn_3_Sn deposited on the MgO (111) (left), schematic of the Hall bar using for transport measurement (right) **b** Room temperature magnetic field dependences of the magnetoconductivity Δσ(H) = (σ(H) − σ(0)). Inset, measurements geometry of the magnetoconductance, blue and red arrow indicate the direction of magnetic field *H* and current *I*, respectively. **c** Room temperature angle dependence of the magnetoconductivity Δσ(θ) at the magnetic field of 8 T. Inset, measurements geometry with the sample rotation axis along [0001], *θ* is the angle between the directions of *H* (blue arrow) and *I* (red arrow).

During the first round of field sweeping measurements, the current *I* is always along the $\left[ 01\bar{1}0 \right]$ direction, while the magnetic field *H* is applied either parallel or transverse to *I*. The room temperature magnetoconductivity, defined as Δσ(*H*) = (σ(*H*) − σ(0)), is plotted in Fig. 4b for a 40 nm Mn_3_Sn sample. As can be seen, when the magnetic field is parallel (transverse) to *I*, the longitudinal (transverse) magnetoconductance increases (decreases) with the increase of *H*. To further clarify the anisotropic character, the angle dependent magnetoconductance measurement (see the inset of Fig. 4c) is performed with *H* = 8 T while rotating the sample from longitudinal ($\left[ 01\bar{1}0 \right]$) to transverse ($[2\bar{1}\bar{1}0]$) direction. The results presented in Fig. 4c are correlated with the angle between the current and magnetic field. Specifically, Δσ reaches a positive maximum value when the *H* is parallel to *I* (*θ* = 0° and 180°), and a negative maximum value when the *H* is perpendicular to *I* (*θ* = 90° and -90°). A small change around the position of *θ* = 0° causes a clear symmetric decrease of the conductance. This large anisotropic magnetoconductance is the transport signature of the chiral anomaly of the Weyl fermion state in Mn_3_Sn. The similar behavior of the bulk crystal samples in Ref. 5 further confirms the excellent noncollinear antiferromagnetic property of our epitaxial thin films.

**Supplementary Note 4**: **Perpendicular magnetic anisotropy of Mn_3_Sn and *β*-Ta based heterostructure device**

The Ni/Co multilayer is stacked on top of Mn_3_Sn by sputtering as a free FM layer to study the generated SOT in Mn_3_Sn. Due to the relatively large surface roughness of the 40 nm Mn_3_Sn film, the thinner 7 nm Mn_3_Sn is selected for the construction of perpendicular magnetic anisotropy (PMA) heterostructures. To induce the perpendicular anisotropy and decouple with the antiferromagnetic Mn_3_Sn, a thin Cu spacer layer of 1nm or 2 nm is inserted in between the Mn_3_Sn and Ni/Co multilayer. The control device, with Mn_3_Sn replaced by the common HM *β*-Ta (denoted as *β*-Ta-based device), is also fabricated using the same process. Figures 5a, 5b, and 5c are the AHE signals from the Mn_3_Sn-based with the inserted two Cu thickness and *β*-Ta-based devices with applied magnetic field sweeping in the normal direction of the film. The vanishingly small signal from the single Mn_3_Sn layer device is also plotted in Fig. 5a for comparison. The almost square AHE loops in both heterostructure devices, undoubtedly confirms that the AHE signal in heterostructures is from the Ni/Co multilayer with well-defined PMA. Therefore, it allows us to use the AHE resistance as a probe for the magnetization direction of Ni/Co multilayer and carry out the current-induced SOT study. The perpendicular magnetic anisotropy field *H_k_* has been also determined to be 1727 Oe, 2487 Oe and 1973 Oe for the Mn_3_Sn (7)/Cu (1)/FM, Mn_3_Sn (7)/Cu (2)/FM and Ta (7)/Cu (2)/FM samples, respectively.

**Figure 5: a** Anomalous Hall loop of Mn_3_Sn (7)/Cu (1)/FM (1.8), the magnetic field dependent Hall resistance of single Mn_3_Sn layer is also shown here. **b** Anomalous Hall loops of Mn_3_Sn (7)/Cu (2)/FM (1.8). **c** Anomalous Hall loops of *β*-Ta (7)/Cu (2)/FM (1.8) PMA heterostructures.

**Supplementary Note 5: Estimate of the out-of-plane polarized spin current by MSHE**

To obtain a more quantitative understanding of the out-of-plane spin current, we adopted the AHE hysteresis loop shift method and estimated the associated SOT effective field^8,9^. The in-plane magnetization along the *x* direction inside of the domain wall experiences an SOT effective field under certain applied current (*J*_C_), which leads to a shift of the *R*_AHE_-*H_z_* hysteresis loop. The shift Δ*H*_z_ is defined as $\Delta H_{z}=H_{center}\left( I^{+} \right)-H_{center}\left( I^{-} \right)$ (see Fig. 6a), where $H_{center}\left( I^{\pm} \right)=\frac{\left[ H_{r}^{+}\left( I^{\pm} \right)-H_{r}^{-}\left( I^{\pm} \right) \right]}{2}$ is the center of the hysteresis loop determined by the difference of positive and negative magnetization-reversal fields $H_{r}^{\pm}\left( I \right)$, and $I^{\pm}$ are positive and negative currents. Δ*H*_z_ thus becomes a direct measure of the SOT effective field $H_{SOT}$ along the *z* direction.

The total effective $H_{SOT}$ can be calculated as $H_{SOT}={(\Delta H}_{z}\left( H_{x}= H_{xsat} \right)-{\Delta H}_{z}\left( H_{x}= H_{-xsat} \right))/2$, where the *H_xsat_* is the field at which ${\Delta H}_{z}$ is saturated. In our case with Mn_3_Sn, both the *y*- and *z*-polarized spin currents can generate a sizable Δ*H*_z_. Specifically, the Δ*H*_z_ generated by *z*-polarized spin current retains a finite value even in the absence of of *H_x_* ^8^, and therefore its associated effective field can be readily determined as$H_{SOT}^{z}={\Delta H}_{z}\left( H_{x}=0 \mathrm{Oe} \right)$.^10^. On the other hand, the Δ*H*_z_ generated by the *y*-polarized spin current only emerges with an assisted field along the *x* direction (*H_x_*). Therefore, the effective SOT field generated purely by the *y*-polarized spin current can be estimated as $H_{SOT}^{y}=H_{SOT}-H_{SOT}^{z}.$

As an example, Fig. 6a (the same as Fig. 2c but the current density is denoted here) shows the *R*_AHE_-*H_z_* hysteresis curves of Mn_3_Sn with *H*_x_ = 0 at *J* = ±4.9×10^6^ A cm^-2^ along the $\left[ 01\bar{1}0 \right]$ direction. As discussed above, the finite shift is solely due to the *z*-polarized spin current. By repeating the measurements for different *J*, the effective SOT field per unit current density for the out-of-plane antidamping torque, which is defined as $\chi_{\sigma_{z}}=H_{SOT}^{z}/J$, can obtained from the slope in Fig. 6b (which is also indicated by the red arrow in Fig. 6c). On the other hand, $\chi_{\sigma_{y}}=H_{SOT}^{y}/J_{c}$ is contributed by the in-plane antidamping torque from the *y*-polarized spin current, which can also be obtained from Fig. 6c. The effective SOT efficiency ($\theta_{zx}^{i}$) can then be calculated using:

$$\theta_{zx}^{i}=\frac{2}{\pi} \frac{{2e\mu}_{0}M_{s}t}{\hbar} \chi_{\sigma_{i}} (1)$$

where $i=z or y$*,* $\hbar$ is the reduced Planck constant, *e* is the electron charge, *μ*_0_ is the vacuum permeability, *M*_s_ = 496 emu cm^-3^ is the saturation magnetization, and *t* = 1.8 nm is thickness of the Ni/Co multilayer. With the obtained $\chi_{\sigma_{y}}$ = 12.8×10^-6^ Oe A^-1^ cm^2^ and $\chi_{\sigma_{z}}$ = 3.9×10^-6^ Oe A^-1^ cm^2^ from Fig. 6c, $\theta_{zx}^{y}$ and $\theta_{zx}^{z}$ are determined to be 0.22 and 0.067, respectively. Using the measured charge conductivity of Mn_3_Sn 367.48 *μ*Ω cm, we obtain the *z*- and *y*-polarized spin conductivities to be $\sigma_{zx}^{y}$ = 6.02×10^4^ [($\hbar$/2e) (Ω m)^-1^] and $\sigma_{zx}^{z}$ = 1.83×10^4^ [($\hbar$/2e) (Ω m)^-1^], respectively, which are slightly smaller than the spin Hall conductivity in CoNiB alloy system^11^ and comparable to these found in previous experiments mentioned in the introduction part. It should be noted that since the insertion of the Cu spacer layer can suppress spin currents^12^, the spin conductivities of Mn_3_Sn are expected to be strongly underestimated. Moreover, in our estimate for simplicity, we do not consider the enhancement of $H_{SOT}^{z}$ by the reorientation of magnetic domains by *H*_x_, which leads to the overestimation of $H_{SOT}^{y}$ and hence the $\sigma_{zx}^{y}$. Therefore, the $\left| \sigma_{zx}^{z}/\sigma_{zx}^{y} \right|$ ratio may be even larger than that estimated above. The sizable $\left| \sigma_{zx}^{z}/\sigma_{zx}^{y} \right|$ ratio is consistent with the recent measurements for a Mn_3_Sn single crystal^13^. This indicates the efficient generation of the *z*-polarized spin current by Mn_3_Sn.

We note that in addition to the MSHE, there might be other contributions to the *z*-polarized spin current in the Mn_3_Sn-based SOT, resulting from interfaces^8,10,14^. However, we expect these contributions play a minor role in the field-free switching we observe. The spin currents driven by interface-related mechanisms are either independent on the magnetic order parameter by symmetry^10,14^, or not strong enough compared to that generated by the bulk effect such as MSHE. For example, it has been suggested that the nonequilibrium *z*-spin polarization can be generated by spin-orbit coupling at low symmetry normal metal (NM)/ferromagnet (FM) interfaces, as reported for Py/WTe_2_ ^14^ and CuPt/CoPt ^10^ systems. This effect is not expected to sizable in our devices, since spin-orbit coupling in both Cu and FM layers is very weak. In addition, the spin polarization generated by this effect cannot be reversed by a magnetic field, and thus cannot explain the change of the switching polarity as shown in Fig. 3 of the main text. The z-polarized spin current due to spin-orbit precession as suggested for a FM/NM/FM trilayer^8,15^, in principle, can appear in our system, since Mn_3_Sn can host a nonvanishing in-plane net magnetic moment and hence can be considered to be a weak ferromagnet. However, the generated out-of-plane spin current in this mechanism is due to the interfacial transmission and reflection^15^, and hence is not expected to be as strong as that generated by a bulk effect such as the MSHE in present study. A good agreement between the *z*-polarized spin current value in our measurements and that in the recent report for a Mn_3_Sn single crystal ^13^ indicates that the SOT switching we observed has largely the bulk origin.


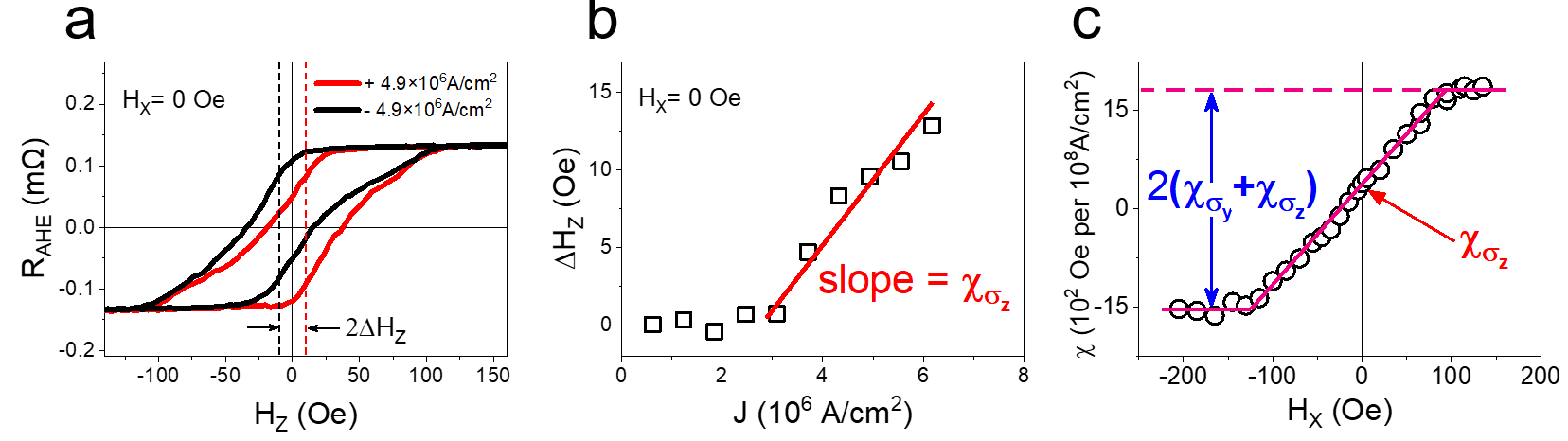


**Figure 6:** **a** *R*_AHE_ vs *H_z_* loop for Mn_3_Sn based sample at *H_x_* = 0 Oe with the applied current density of ±4.9×10^6^ A cm^-2^. **b** The loop shift Δ*H_z_* for the sample under different applied current density at *H_x_* = 0 Oe. **c** The slope of $\chi$ = Δ*H*_z_/*J* at different *H_x_* values.

**Supplementary Note 6**: **Exchange bias effect of Mn_3_Sn based heterostructure**

To determine the exchange bias effect of Mn_3_Sn(7)/Cu(1)/[Ni/Co]_3_/(1.8) and Mn_3_Sn(7)/Cu(2)/[Ni/Co]3(1.8), the out-of-plane anomalous Hall loops were measured at different temperatures. As shown in Figs. 7a and 7b, the magnitude of coercivity monotonously increases as the temperature cooling down from 300 K to 10 K for 2 samples. Meanwhile the exchange bias field keep at almost zero until the temperature reach sufficiently low, *i.e.* 100 K for Mn_3_Sn(7)/Cu(1)/[Ni/Co]_3_/(1.8) sample and 25 K for Mn_3_Sn(7)/Cu(2)/[Ni/Co]_3_/(1.8). Figure 7c summarizes temperature dependence of *H*_EB_ of these two samples, which is in line with our perception that thicker inserting copper layer will more effectively decouple the exchange coupling between antiferromagnetic Mn_3_Sn and ferromagnetic layer. Observable exchange bias only appears at sufficient low temperature with enhanced exchange coupling. This result unambiguously excludes the exchange bias as the possible reason to explain the field free switching behavior of our Mn_3_Sn based sample at room temperature. Meanwhile, the exchange bias at low temperature also proves the antiferromagnetic nature of our Mn_3_Sn thin film.

**Figure 7: a,b** Temperature dependence of exchange bias and coercivity of Mn_3_Sn (7)/Cu(2)/[Ni/Co]_3_/ (1.8) (**a**) Mn_3_Sn(7)/Cu(1)/[Ni/Co]_3_/ (1.8) (**b). c** Comparison of exchange bias for the 2 samples.

**Supplementary Note 7**: **SOT switching results for I//H//Mn_3_Sn**$\boldsymbol{[2}\bar{\boldsymbol{1}}\bar{\boldsymbol{1}}\boldsymbol{0]}$ **and I//Mn_3_Sn**$\left[ \boldsymbol{01}\bar{\boldsymbol{1}}\boldsymbol{0} \right]$**, H//Mn_3_Sn**$\boldsymbol{[2}\bar{\boldsymbol{1}}\bar{\boldsymbol{1}}\boldsymbol{0}$**]**

According to the DFT calculation, the *z*-spin component can also be realized when the current is applied along Mn_3_Sn $[2\bar{1}\bar{1}0]$. Indeed, no obvious shift of the loop is observed with *I* = + (-) 4 mA as shown in Fig. 8a. When the current increases to +16 mA or −16 mA (Fig. 8b), a sizable positive or negative shift occurs. Again, the threshold current to introduce the shift is about 10 mA (Fig. 8c), above which the shift increases almost linearly with the increase of *I*. The maximum shift value with *I* = 20 mA is smaller as compared to the case in the main text, which is consistent with the slightly lower field-free switching ratio that will be presented shortly.

Magnetization switching can be also realized without any external magnetic field when the pulse current is applied along Mn_3_Sn $[2\bar{1}\bar{1}0]$ direction, as shown in Fig. 8d, the switching ratio is about 48%, which is slightly smaller than the case that the current is applied along $\left[ 01\bar{1}0 \right]$. Figures 8e shows the switching ratio dependence of different magnetic field along $[2\bar{1}\bar{1}0]$ direction, the dependence on the external assistive field is similar to Fig. 3c of main manuscript. The difference is that the vanished switching appears at around +7 Oe. We presume that the this is determined by the preferred AFM domains (AFM1 or AFM2), which leads to a positive or negative $\sigma_{zx}^{z}$, as shown in Fig. 1d of the main text.

**Figure 8: a, b** *R*_AHE_ Vs. *H*_z_ curve when the detecting currents are ±4 mA and ±16 mA. **c** Δ*H*_z_ Vs. *I*, the critical *I* to shift AHE curve is about 10 mA, +*I* will shift the AHE curve to the -*x* while -*I* lead to the opposite shift. **d** Current induced magnetization switching with anticlockwise polarity with the absence of an external magnetic field. **e** The evolution of the switching polarity and switching portion under different magnetic field, here the direction of magnetic field is in-plane and parallel to the current.

We have also measured the SOT switching when the current is applied along the *x* ($\left[ 01\bar{1}0 \right]$) direction and the external magnetic field along the *y* ($[2\bar{1}\bar{1}0]$) direction. Due to the magnetic spin Hall conductivity $\sigma_{zx}^{z}$, which is related to the field-free switching of the device, being finite in the AFM1 configuration but absent in the AFM2 configuration by symmetry (Table 1), an out-of-plane spin current is expected to gradually decrease to zero in this case as *H_y_* favors the AFM2 domains and suppress the AFM1 domains. Therefore, we expect that *H_y_* will suppress the switching but won’t influence the polarity, since it only reduces the *z*-polarized spin current but does not reverse its spin polarization. This is indeed has been confirmed in our measurements, as seen from Fig. 9.


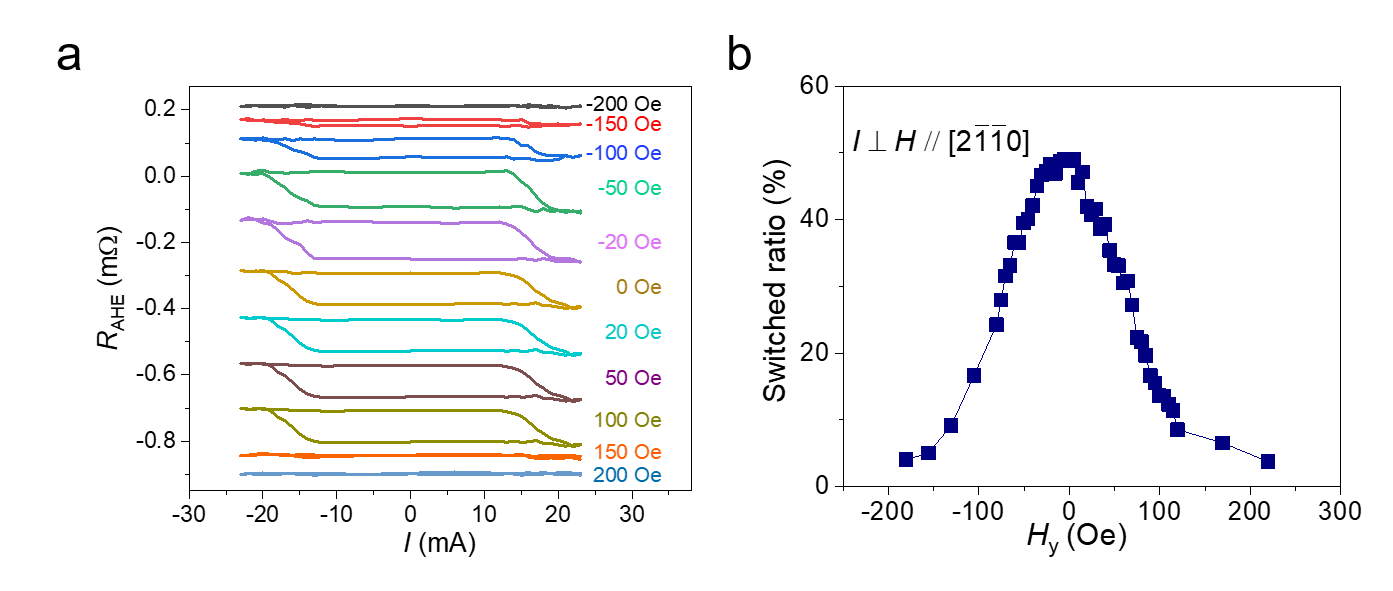


**Figure 9: a** The switching curve under different external magnetic fields along the $[2\bar{1}\bar{1}0]$ direction with *I* being along the $\left[ 01\bar{1}0 \right]$ direction. **b** The switching ratio indicating the potion of the switched AFM domains as a function of magnetic field *H_y_*.

**Supplementary Note 8**: **Evaluation of Joule heating effect and other control experiments**

Joule heating has been carefully evaluated in our studies. To determine the actual temperature during the switching measurement, we first measured the *R*_xx_-Pulse current amplitude curve as shown in Fig. 10a. For comparison, temperature dependence of *R*_xx_ was also measured from 300 K to 100 K as shown in Fig. 10b. The linear fitting provides the quantitative relationship between *R*_xx_ and temperature, so we can estimate the actual temperature with each electric current by converting *R*_xx_ into actual temperature in Fig. 10c. We can hence estimate the actual device temperature to be ~340 K for the maximum current used in Fig. 2c of main manuscript and ~360 K for the critical switching current used in Fig. 3a of main manuscript, which are well below the *T_N_* of Mn_3_Sn. This eliminates Joule heating as the possible origin of the observed effect.

**Figure 10:** **a** Resistance of Mn_3_Sn (7)/Cu (1)/FM (1.8) device *vs.* pulse current amplitude. (Current pulse width = 800 μs) **b** Linear fitting curve for the temperature dependence of device resistance. **c** Determination of device temperature with different pulse amplitudes.

We have also evaluated the temperature effect on the spin torque by measuring the harmonic anomalous Hall loops at elevated temperatures. It is well established that the peak amplitude of the second harmonic anomalous Hall loop is proportional to the strength of spin torque. The results are shown in Fig. 11. The first harmonic anomalous Hall loops in Fig. 11a demonstrate good perpendicular magnetic anisotropy of the device at various temperatures. On other hand, in Fig. 11b, one can see that the shape of the second harmonic Hall loop is largely preserved at around 360 K. Upon a further increase of temperature toward 420 K, the second harmonic Hall loop shows much smaller signal compared to the one at 298 K. The results in Fig. 11 indicate that the Mn_3_Sn spin texture and its associated spin torque are maintained at 360 K. Upon quenching of spin texture by elevating temperature up to the Mn_3_Sn Neel temperature, the spin torque generated by Mn_3_Sn is largely suppressed.


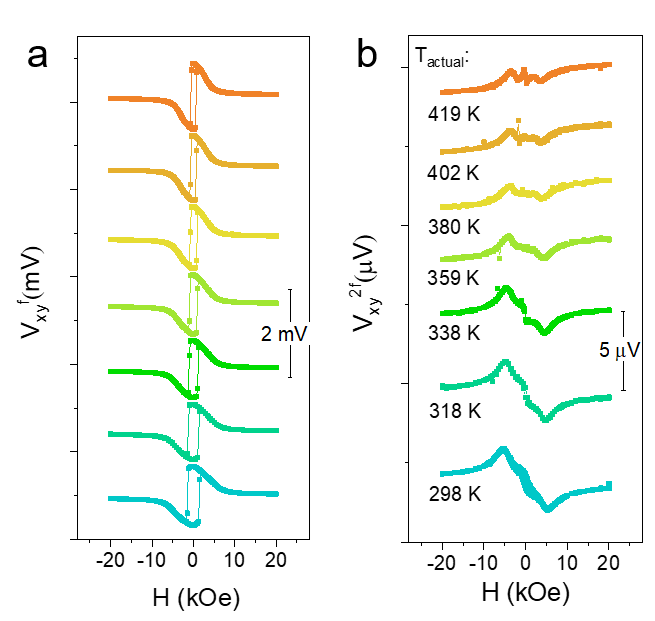


**Figure 11:** The first harmonic (**a**) and second harmonic (**b**) anomalous Hall loops measured at various temperatures for the Mn_3_Sn sample. An ac current with a current density of 1×10^7^ A/cm^2^ and a frequency of 13.7 Hz is employed for the measurements. The actual device temperature *T*_actual_ is determined by the temperature dependence of the device resistance and indicated at each second harmonic anomalous Hall loop in **b**.

Moreover, to prove the shift of the *R*_AHE_-*H*_z_ hysteresis loop in Fig. 2c is due to *z*-polarized spin component generated by Mn_3_Sn and to eliminate potential spurious effects caused by Joule heating, a referenced measurement was conducted using *β*-Ta based heterostructure, as shown in Figs. 12a, b. Similarly, no shift of the loop was seen when the applied amplitude of *I* is at 4 mA and 34 mA (the effect current density in *β*-Ta is about 1.4×10^7^ A cm^-2^). This indicates that Joule heating is not responsible for the *R*_AHE_-*H*_z_ hysteresis loop shift in Fig. 2c. In addition, current induced switching at zero magnetic field was performed in *β*-Ta based heterostructure as well. As shown in Fig. 12c, no magnetism switching was observed thus confirm once again that *z*-polarized spin is the main mechanism of current induced magnetization switching in the absence of external field.

**Figure 12: a** *R*_AHE_ vs. *H*_z_ curve when the bias currents are ±4 mA and ±34 mA **b** *R*_AHE_ vs. *J*_c_ in the absence of an external magnetic field for the *β*-Ta based heterostructure.

To elucidate the influence of the inserted Cu layer on the SOT device performance, we have prepared a Ta-based SOT device by adding a Ta layer directly on top of the ferromagnet. The stack structure is Ta (3 nm)/Cu (2 nm)/Co-Ni (1.8 nm)/Ta (4 nm)/TaO_x_ (2 nm) and a good perpendicular magnetic anisotropy with *H*_K_ = 1850 Oe is ensured by the Cu layer below the FM layer. As seen from Figure 13, the switching current of 9.2×10^6^ A/cm^2^ at 300 K in this device is still significantly larger than that in the Mn_3_Sn-based device with the Cu spacer (Fig. 4b). These results eliminate a possible concern that the different spin mixing conductance at the *β*-Ta/Cu and Mn_3_Sn/Cu interfaces is responsible for a much more efficient performance of our Mn_3_Sn based device compared to the conventional SOT devices.


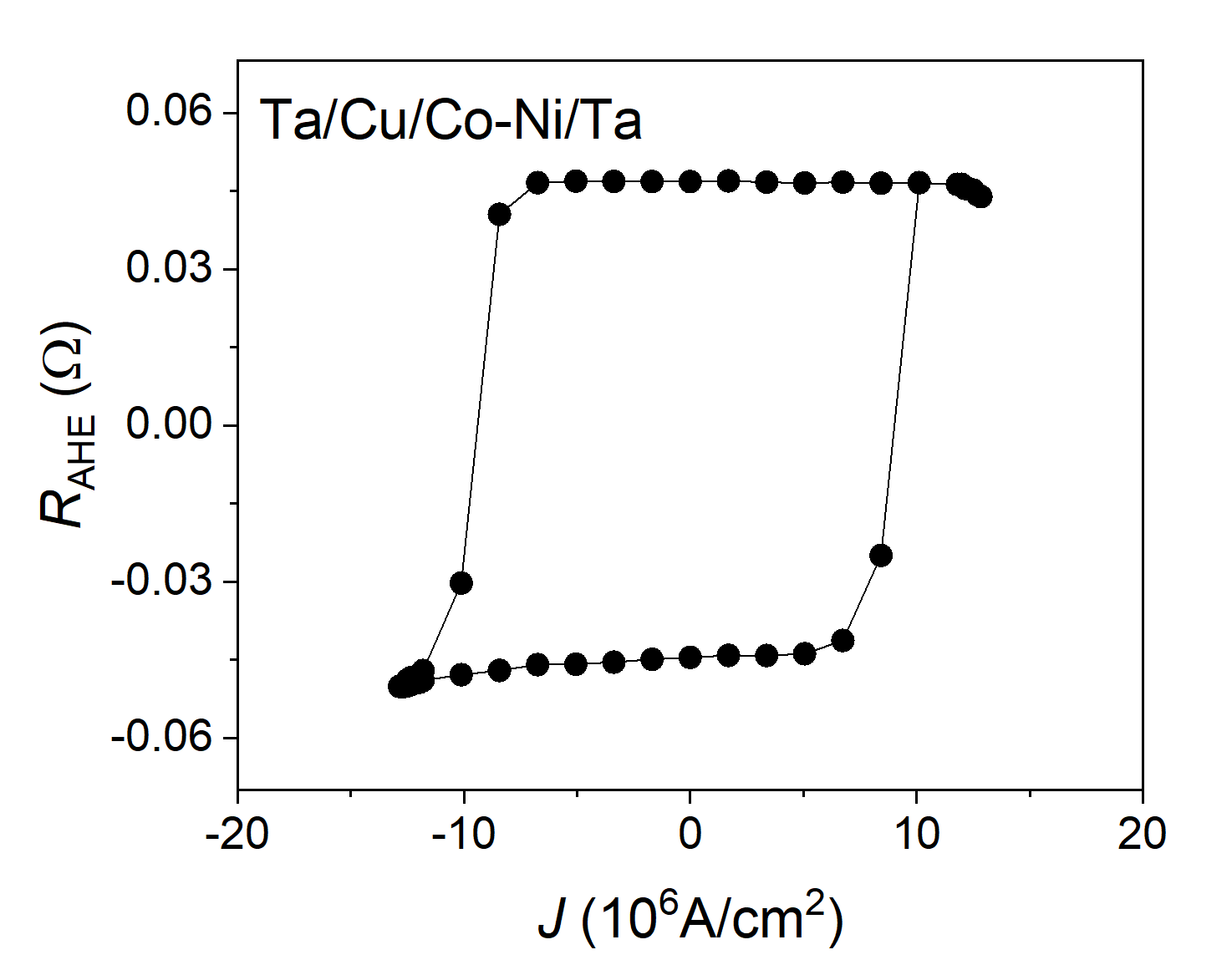


**Figure 13:** SOT switching curve for the Ta (3 nm)/Cu (2 nm)/Co-Ni (1.8 nm)/Ta (4 nm)/TaO_x_ (2 nm) device. A 300 Oe assisted field is applied along current direction.

**Supplementary Note 9: Magnetization switching by MSHE in a 100 nm device**

The MSHE driven magnetization switching has been examined in a nanostructured device. The nanowire device was fabricated with a lateral width of 100 nm using e-beam lithography and ion-milling. Fig. 14a shows that the device exhibits perpendicular magnetic anisotropy, as seen from the nearly square loop of the *R*_AHE_-*H*_z_ curve. However, we have not been able to realize the field-free switching in this device (Fig. 14b). We consider two possible reasons:

1) Due to the width scaling, the anisotropy and thus coercivity of the Ni/Co multilayer increase dramatically. Specifically, the coercivity rises from 109 Oe in the microwire to 561 Oe in the nanowire. This leads to a much larger critical current density required for switching^16,17^.

2) With the much smaller size of the device, the inevitable Joule heating at a larger current strongly suppresses the imbalance of the magnetic domains, resulting in a small net *z*-polarized spin current, which is not sufficient for switching.

At the same time, a clear switching has been observed when an external field as small as *H_x_* = 5 Oe was applied, due to the reorientation of the magnetic domains by *H_x_*. Although the anomalous Hall response induced by the MSHE switching (Fig. 14b) is a factor of 3 weaker than that induced by the magnetic field switching (Fig. 14a) due to the possible reasons discussed above, it still indicates that a field free switching with a large switching ratio in a Mn_3_Sn based nanoscale SOT device can be eventually realized if the magnetic domains of Mn_3_Sn are well orientated and pinned under the application of current. This may be realized by depositing Mn_3_Sn on a hard ferromagnetic substrate with an in-plane anisotropy. In this case, due to a small but nonvanishing net magnetic moment of Mn_3_Sn, the magnetic domains are supposed to be aligned by the strong interfacial exchange bias field. Another direction to solve this problem is to remove the Cu spacer which suppresses the spin current in Mn_3_Sn based SOT devices.

**Figure 14**: **a** Anomalous Hall loop of the 100 nm nanowire device. **b** SOT switching curves with and without the assistive field along *x*-axis at 300 K.

**Supplementary Note 10: Macro-spin simulation of MSHE driven magnetization switching**

In order to further demonstrate the superiority of the switching by MSHE, we performed macro-spin simulation of a SOT device with different $\sigma_{zx}^{z}/\sigma_{zx}^{y}$ ratios. The single domain simulation is implemented by numerically solving SOT modified LLG equation with damping-like torque and field-like torque^18^:

$\frac{\partial\boldsymbol{m}}{\partial t}=-\gamma\mu_{0}\boldsymbol{m}\times\boldsymbol{H}_{\boldsymbol{eff}}+\alpha\boldsymbol{m}\times\frac{\partial\boldsymbol{m}}{\partial t}+\frac{\gamma\hbar c^{\parallel}J_{e}}{2eM_{s}t_{FM}}\left( \boldsymbol{m}\times\boldsymbol{\sigma}\times\boldsymbol{m} \right)+\frac{\gamma\hbar c^{\perp}J_{e}}{2eM_{s}t_{FM}}\left( \boldsymbol{\sigma}\times\boldsymbol{m} \right)$ (2)

where **m** is the unit vector along the magnetization of free layer, which can be written as $\left( cos\phi_{m}sin\theta_{m},sin\phi_{m}sin\theta_{m},cos\theta_{m} \right)$. **σ** is the unit vector along spin polarization, *J_e_* is the current density. Effective field $H_{ani}\left( cos\theta_{m} \right)\boldsymbol{z+}\boldsymbol{H}_{\mathbf{ext}}$, where the anisotropy field $H_{ani}$= 3500 Oe, $\boldsymbol{H}_{\mathbf{ext}}$ is the external field. Gilbert damping constant *α* = 0.03, spin Hall angle $c^{\parallel}$= 0.3 and $c^{\perp}$=0, saturation magnetization *M*_s_ = 800 emu/cc, free layer thickness *t*_FM_ *=* 1.8 nm. The current pulse-width is set as 100 ns while plotting trajectories of **m**, and set as 2 ns in other simulations. current rise/fall time = 0.2 ns, relaxation time = 100 ns.


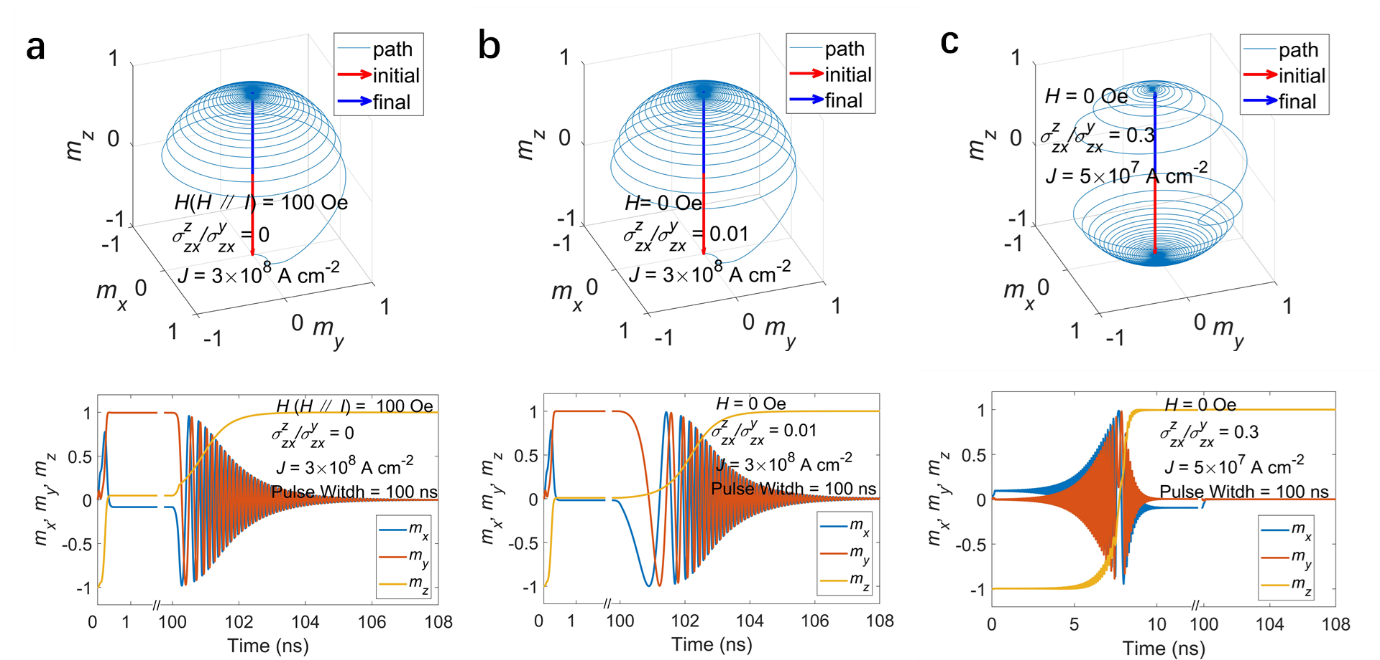


**Figure 15:** Simulated magnetization switching trajectories with conventional *y*-polarized spin current switching at **a** $\sigma_{zx}^{z}/\sigma_{zx}^{y}$ = 0, *H_x_* = 100 Oe; and *z*-polarized spin current switching at **b** $\sigma_{zx}^{z}/\sigma_{zx}^{y}$ = 0.01, *H_x_* = 0 Oe; and **c** $\sigma_{zx}^{z}/\sigma_{zx}^{y}$ = 0.3, *H*_x_ = 0 Oe.

We find that the field-free switching can be realized for all nonzero $\sigma_{zx}^{z}/\sigma_{zx}^{y}$ ratios (Figs. 15). For a small $\sigma_{zx}^{z}/\sigma_{zx}^{y}$ ratio, the switching trajectory with zero *H_x_* field is similar to the case of the conventional SOT switching with an assisted *H_x_* field (Figs. 15a,b), where the magnetic moment (initially pointing along the -*z* direction) is first pulled toward the *y* direction by the torque $\sim m\times(m\times y)$exerted by the *y*-polarized spin current from the conventional SHE, and then relaxes to the +*z* direction. There is no precession during the application of the current, indicating the torque is majorly $\sim m\times(m\times y)$ generated by $\sigma_{zx}^{y}$which directly competes with the precession. These trajectories indicate that when $\sigma_{zx}^{z}/\sigma_{zx}^{y}$ is small, the *z*-polarized spin current generated by the MSHE only contributes to the symmetry breaking as the assisted field does. On the other hand, when $\sigma_{zx}^{z}/\sigma_{zx}^{y}$is not too small, the magnetic moment is directly switched to the opposite direction during the application of the current, and the precession is well maintained during the application of the current (Fig. 15c). This is a typical characteristic of the switching by the out-of-plane antidamping torque $\sim m\times\left( m\times z \right)$ due to the *z*-polarized spin current. Since the switching driven by the out-of-plane antidamping torque does not compete with the precession, the critical current for such switching is much smaller than that in a conventional SOT switching, and it further decreases with the increase of $\sigma_{zx}^{z}/\sigma_{zx}^{y}$ (Fig. 16).

Therefore, based on the sizable $\sigma_{zx}^{z}/\sigma_{zx}^{y}$ estimated above and the low critical switching current observed in our field-free switching, we argue that the field-free switching we observed is dominated by the out-of-plane antidamping torque driven by the *z*-polarized spin current resulting from the MSHE in Mn_3_Sn.


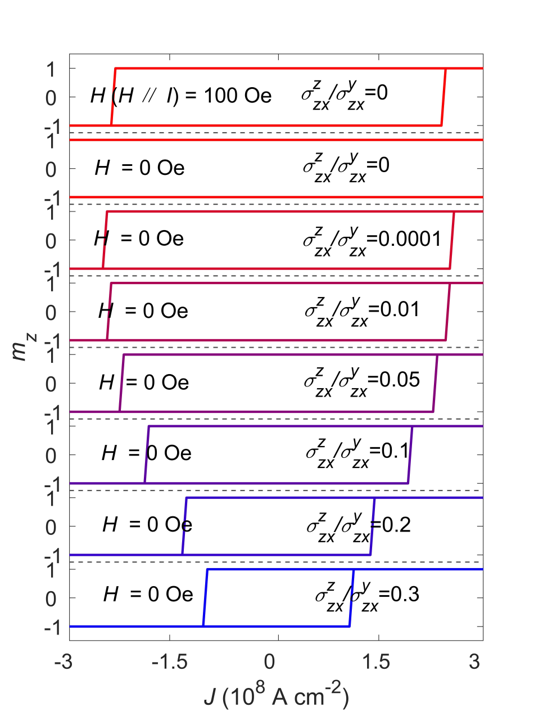


**Figure 16:** Simulated *m_z_* *vs* *J* switching curves for the conventional SOT switching with $\sigma_{zx}^{z}/\sigma_{zx}^{y}$ = 0 at *H_x_* = 0 or 100 Oe, and the *z*-polarized spin current switching with different $\sigma_{zx}^{z}/\sigma_{zx}^{y}$ ratios at *H_x_* = 0.

**Supplementary Note 11: Thermal effects during MSHE driven magnetization switching**

In view of the Joule heating rising the device temperature (Supplementary Note 8), additional micromagnetic simulations are performed by including the thermal effects. A Gaussian-distributed random thermal fluctuation field *H_th_* with mean = 0 and standard deviation = $\sqrt{\frac{2\alpha k_{B}T}{\gamma M_{s}V\delta t}}$ is added into the Supplementary Eq. (2), where *k_B_* is the Boltzmann constant, *T* is the temperature, *V* is the volume of a ferromagnet taken to be 50 nm × 50 nm × 1.8 nm, $\gamma$ is the gyromagnetic ratio, and $\delta t$ is the integration time step^19^. First, for a pure *y*-polarized spin current, *i.e.* $\sigma_{zx}^{z}/\sigma_{zx}^{y}$ = 0, we find that the switching is never achieved in the absence of an in-plane assisted magnetic field *H_x_*, even with a strong thermal assistance. This indicates that the thermal effect alone cannot result in a field-free switching of the perpendicular magnetization. Second, we compare the switching behaviors at 300 K and 370 K in the presence of either finite in-plane field *H_x_* _­_or finite $\sigma_{zx}^{z}$ for pulse widths in the range of 1 ns to 1000 ns. As an example, we show in Fig. 17 the switching trajectory in the presence of a long pulse width of 1000 ns. Except additional noises present, including the thermal fluctuation field does not produce any qualitative effect on the magnetization switching trajectory (compare Figs. 15 and 17). The magnetization switching trajectory with other tested pulse widths demonstrate similar behavior.


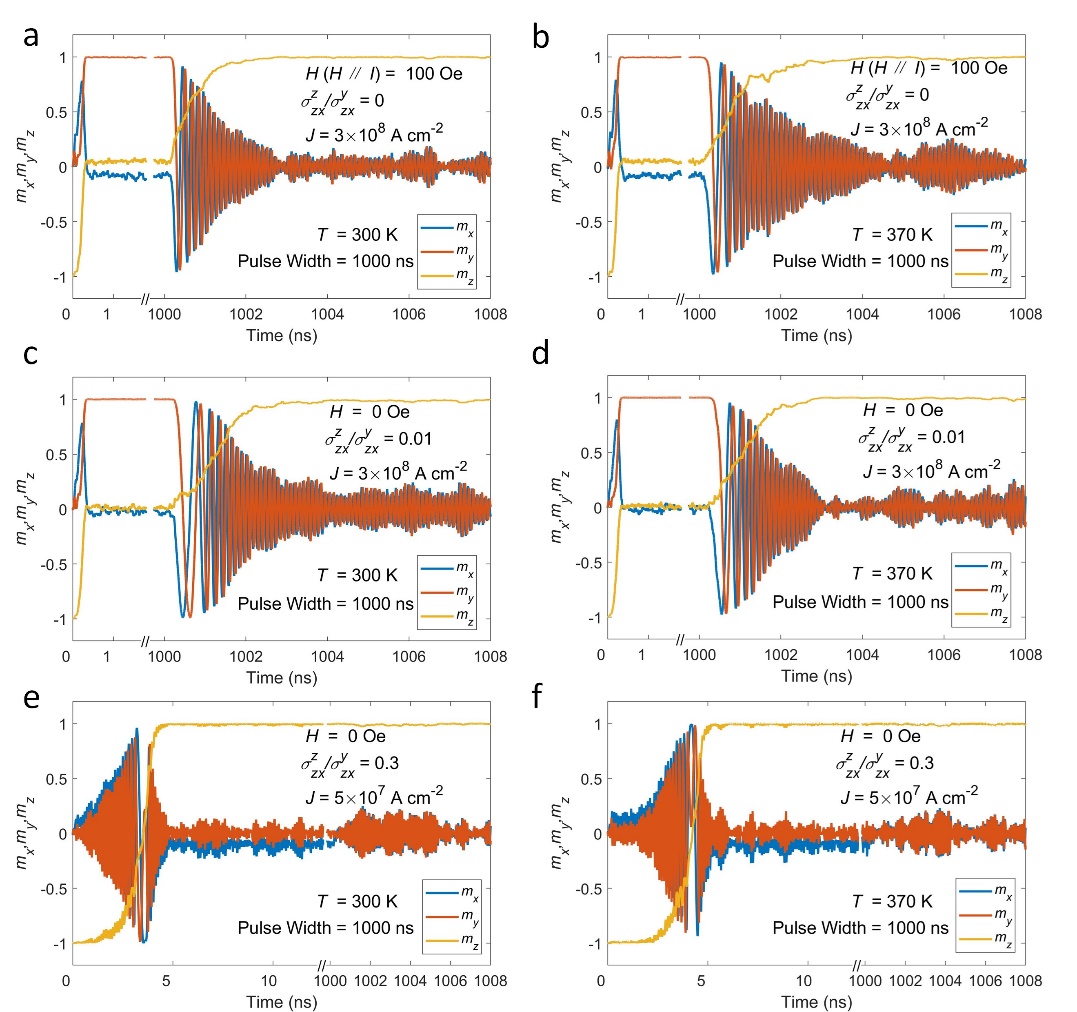


**Figure 17:** Simulated magnetization switching trajectories after considering the thermal fluctuation field with conventional *y*-polarized spin current switching **a** and **b** $\sigma_{zx}^{z}/\sigma_{zx}^{y}$ = 0, *H_x_* = 100 Oe at 300 K and 370 K, respectively; and *z*-polarized spin current switching **c** and **d** $\sigma_{zx}^{z}/\sigma_{zx}^{y}$ = 0.01, *H_x_* = 0 Oe at 300 K and 370 K, respectively; **e** and **f** $\sigma_{zx}^{z}/\sigma_{zx}^{y}$ = 0.3, *H_x_* = 0 Oe at 300 K and 370 K, respectively.

Fig. 18 summarizes the critical switching current with different pulse widths for $\sigma_{zx}^{z}/\sigma_{zx}^{y}$ = 0, *H_x_* = 100 Oe (Fig. 18a) and $\sigma_{zx}^{z}/\sigma_{zx}^{y}$ = 0.3, *H_x_* = 0 Oe (Fig. 18b) at 300 K and 370 K. In both cases, the switching current density increases at short pulses, similar to the previous work^20^. Moreover, we find that the thermally assisted switching plays a more important role in the case of *z*-polarized spin current than in the case of *y*-polarized spin current, as is evident from the larger increase of the critical current density with the decreasing pulse width. We note, however, that, in terms of the switching efficiency, the *z*-polarized spin current is superior than the *y*-polarized spin current due to a much lower current amplitude required for switching in the entire simulated range. In addition, by comparing the results at 300 K and 370 K, the almost overlapped curves in Fig. 18b suggest that the *z*-polarized spin current is more immune to the temperature increase induced by Joule heating. Based on this additional analysis, we conclude that neither the reduced pulse width nor the thermal effects impact our main result emphasizing the efficient field-free switching of the perpendicular magnetization driven by the *z*-polarized spin current generated by a magnetic spin Hall effect.


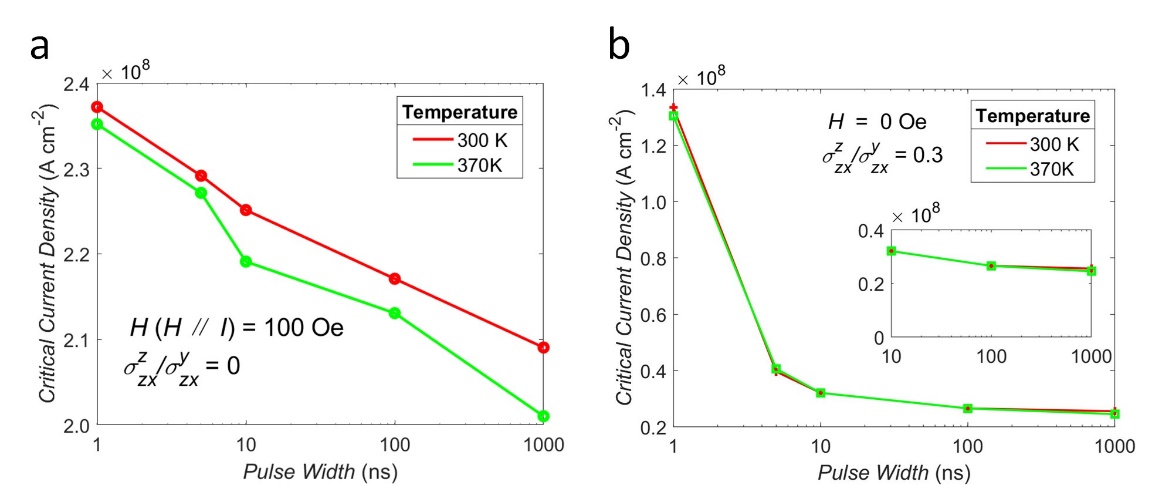


**Figure 18:** Simulated critical current density at 300 K and 370 K with **a** conventional *y*-polarized spin current switching ($\sigma_{zx}^{z}/\sigma_{zx}^{y}$ = 0, *H_x_* = 100 Oe) and **b** *z*-polarized spin current switching ($\sigma_{zx}^{z}/\sigma_{zx}^{y}$ = 0.3, *H_x_* = 0 Oe).

**References**

1. Nakatsuji, S., Kiyohara, N. & Higo, T. Large anomalous Hall effect in a non-collinear antiferromagnet at room temperature. *Nature* **527**, 212 (2015).

2. Tomiyoshi, S. & Yamaguchi, Y. Magnetic structure and weak ferromagnetism of Mn3Sn studied by polarized neutron diffraction. *Journal of the Physical Society of Japan* **51**, 2478 (1982).

3. Brown, P., Nunez, V., Tasset, F., Forsyth, J. & Radhakrishna, P. Determination of the magnetic structure of Mn_3_Sn using generalized neutron polarization analysis. *Journal of Physics: Condensed Matter* **2**, 9409 (1990).

4. Nagamiya, T., Tomiyoshi, S. & Yamaguchi, Y. Triangular spin configuration and weak ferromagnetism of Mn_3_Sn and Mn_3_Ge. *Solid State Communications* **42**, 385 (1982).

5. Kuroda, K., Tomita, T., Suzuki, M. T., Bareille, C., Nugroho, A. A., Goswami, P., Ochi, M., Ikhlas, M., Nakayama, M., Akebi, S., Noguchi, R., Ishii, R., Inami, N., Ono, K., Kumigashira, H., Varykhalov, A., Muro, T., Koretsune, T., Arita, R., Shin, S., Kondo, T. & Nakatsuji, S. Evidence for magnetic Weyl fermions in a correlated metal. *Nature Materials* **16**, 1090 (2017).

6. Zhang, Y., Sun, Y., Yang, H., Zelezny, J., Parkin, S. P. P., Felser, C. & Yan, B. H. Strong anisotropic anomalous Hall effect and spin Hall effect in the chiral antiferromagnetic compounds Mn_3_X (X = Ge, Sn, Ga, Ir, Rh, and Pt). *Physical Review B* **95**, 075128 (2017).

7. Yang, H., Sun, Y., Zhang, Y., Shi, W.-J., Parkin, S. S. P. & Yan, B. Topological Weyl semimetals in the chiral antiferromagnetic materials Mn_3_Ge and Mn_3_Sn. *New Journal of Physics* **19**, 015008 (2017).

8. Baek, S. C., Amin, V. P., Oh, Y. W., Go, G., Lee, S. J., Lee, G. H., Kim, K. J., Stiles, M. D., Park, B. G. & Lee, K. J. Spin currents and spin-orbit torques in ferromagnetic trilayers. *Nature Materials* **17**, 509 (2018).

9. Pai, C. F., Mann, M., Tan, A. J. & Beach, G. S. D. Determination of spin torque efficiencies in heterostructures with perpendicular magnetic anisotropy. *Physical Review B* **93**, 144409 (2016).

10. Liu, L., Zhou, C., Shu, X., Li, C., Zhao, T., Lin, W., Deng, J., Xie, Q., Chen, S., Zhou, J., Guo, R., Wang, H., Yu, J., Shi, S., Yang, P., Pennycook, S., Manchon, A. & Chen, J. Symmetry-dependent field-free switching of perpendicular magnetization. *Nature Nanotechnology* **16**, 277 (2021).

11. Hibino, Y., Taniguchi, T., Yakushiji, K., Fukushima, A., Kubota, H. & Yuasa, S. Large Spin-Orbit-Torque Efficiency Generated by Spin Hall Effect in Paramagnetic Co-Ni-B Alloys. *Physical Review Applied* **14**, 064056 (2020).

12. Fan, X., Celik, H., Wu, J., Ni, C. Y., Lee, K. J., Lorenz, V. O. & Xiao, J. Q. Quantifying interface and bulk contributions to spin-orbit torque in magnetic bilayers. *Nature Communications* **5**, 3042 (2014).

13. Kondou, K., Chen, H., Tomita, T., Ikhlas, M., Higo, T., MacDonald, A. H., Nakatsuji, S. & Otani, Y. Giant field-like torque by the out-of-plane magnetic spin Hall effect in a topological antiferromagnet. *Nature Communications* **12**, 6491 (2021).

14. MacNeill, D., Stiehl, G. M., Guimaraes, M. H. D., Buhrman, R. A., Park, J. & Ralph, D. C. Control of spin–orbit torques through crystal symmetry in WTe_2_/ferromagnet bilayers. *Nature Physics* **13**, 300 (2016).

15. Amin, V. P., Zemen, J. & Stiles, M. D. Interface-Generated Spin Currents. *Physical Review Letters* **121**, 136805 (2018).

16. Zhang, C., Fukami, S., Sato, H., Matsukura, F. & Ohno, H. Spin-orbit torque induced magnetization switching in nano-scale Ta/CoFeB/MgO. *Applied Physics Letters* **107**, 012401 (2015).

17. Jinnai, B., Sato, H., Fukami, S. & Ohno, H. Scalability and wide temperature range operation of spin-orbit torque switching devices using Co/Pt multilayer nanowires. *Applied Physics Letters* **113**, 212403 (2018).

18. Legrand, W., Ramaswamy, R., Mishra, R. & Yang, H. Coherent Subnanosecond Switching of Perpendicular Magnetization by the Fieldlike Spin-Orbit Torque without an External Magnetic Field. *Physical Review Applied* **3**, 064012 (2015).

19. Lee, D.-K. & Lee, K.-J. Spin-orbit Torque Switching of Perpendicular Magnetization in Ferromagnetic Trilayers. *Scientific Reports* **10**, 1772 (2020).

20. Lee, K.-S., Lee, S.-W., Min, B.-C. & Lee, K.-J. Thermally activated switching of perpendicular magnet by spin-orbit spin torque. *Applied Physics Letters* **104**, 072413 (2014).
